# Physico-chemical properties and catalytic activity of the sol-gel prepared Ce-ion doped LaMnO_3_ perovskites

**DOI:** 10.1038/s41598-019-44118-1

**Published:** 2019-05-23

**Authors:** Anees A. Ansari, Naushad Ahmad, Manawwer Alam, Syed F. Adil, Shahid M. Ramay, Abdulrahman Albadri, Ashfaq Ahmad, Abdullah M. Al-Enizi, Basel F. Alrayes, Mohamed E. Assal, Abdulrahman A. Alwarthan

**Affiliations:** 1 King Abdullah Institute for Nanotechnology, King Saud University, Riyadh, 11451 Saudi Arabia; 2 Department of Chemistry, College of Sciences, King Saud University, Riyadh, 114551 Saudi Arabia; 3 Department of Physics & Astrophysics, College of Sciences, King Saud University, Riyadh, 114551 Saudi Arabia; 4 National Center for Nanotechnology and Advanced Materials, King Abdulaziz City for Science and Technology, Riyadh, 11442 Saudi Arabia; 5 Central Laboratory, College of Science, King Saud University, Riyadh, 11451 Saudi Arabia

**Keywords:** Organocatalysis, Catalyst synthesis

## Abstract

Ce-doped LaMnO_3_ perovskite ceramics (La_1−x_Ce_x_MnO_3_) were synthesized by sol-gel based co-precipitation method and tested for the oxidation of benzyl alcohol using molecular oxygen. Benzyl alcohol conversion of ca. 25–42% was achieved with benzaldehyde as the main product. X-ray diffraction (XRD), thermogravimetric analysis (TGA), BET surface area, transmission electron microscopy (TEM), X-ray photoelectron spectroscopy (XPS), temperature-programmed reduction (H_2_-TPR), temperature-programmed oxidation (O_2_-TPO), FT-IR and UV-vis spectroscopic techniques were used to examine the physiochemical properties. XRD analysis demonstrates the single phase crystalline high purity of the perovskite. The Ce-doped LaMnO_3_ perovskite demonstrated reducibility at low-temperature and higher mobility of surface O_2_-ion than their respective un-doped perovskite. The substitution of Ce^3+^ ion into the perovskite matrix improve the surface redox properties, which strongly influenced the catalytic activity of the material. The LaMnO_3_ perovskite exhibited considerable activity to benzyl alcohol oxidation but suffered a slow deactivation with time-on-stream. Nevertheless, the insertion of the A site metal cation with a trivalent Ce^3+^ metal cation led to an enhanced in catalytic performance because of atomic-scale interactions between the A and B active site. La_0.95_Ce_0.05_MnO_3_ catalyst demonstrated the excellent catalytic activity with a selectivity of 99% at 120 °C.

## Introduction

Presently, perovskite-based materials are gaining immense popularity in the field of material science due to their extraordinary optical, electro-magnetic properties. Perovskite materials mostly applied for removing common exhaust pollutants including carbon monoxide, hydrocarbon, ammonia oxidation, water dissociation, and NOx, etc.^1–3^. Amongst different perovskite, Mn-containing oxide materials have been growing a considerable interest from the researchers because of the large specific external area, high thermo-chemical durability and extraordinary catalytic performance even at environmental conditions^2–9^. These excellent physicochemical properties of Mn-based perovskite materials made them an ideal candidate for their applications in the decomposition of customary use pollutants including carbon monoxide, NOx, and poisonous hydrocarbons. In this regard, various types of catalytic conversion technologies were developed^4,5,8,10,11^. Besides that, in order to make the catalytic combustion widely applicable, the development of reliable technologies is highly desirable. Amongst various catalytic active perovskite materials, lanthanide (Ln^3+^) ion substituted perovskite demonstrated superior activities^4–8,10,12^. Such materials revealed higher catalytic activity and superior thermal stability for hydrocarbon combustion than their respective un-substituted perovskites^2,7,10^.

Owing to the outstanding catalytic activity of perovskite-type oxide ABO_3_, where A is 12 coordinated and larger cation in size, whereas B is 6 fold coordination and smaller cation in size with oxygen anion. The partial co-doping of the A-site by the transition metal ions with dissimilar valance generate a structural defect because of bond stretching and amend the valence of the B-site to meet the chemical charge balance of the perovskite structure; actually, it is the prime origin for extraordinary catalytic oxidation performance of the ABO_3_ based oxides. Therefore, doping of similar valence state ions at A or B sites might be altered the crystal structure, geometrical symmetry and disturb the oxidation states of the cations without altering the structure. Besides that, the variation of Mn^4+/^Mn^3+^ ratio has the main effect on the catalytic activities of ABO_3_ materials. The partial doping of Ce^3+^ ion into LaMnO_3_ altered the catalytic activity because of an increase in specific surface area, surface defects, oxygen mobility, and redox ability. Ceria has the capability to absorb and release the oxygen vacancies, and these oxygen species play a crucial role in the overall catalytic activities of the CeO_2_-based perovskites^13–18^. Owing to the oxidation state transformation behavior of ceria between Ce^3+^ and Ce^4+^ dependent on the O_2_ partial pressure in the nearby atmosphere^13,14^. Usually, the redox behavior of Ce^3+^ is determined by morphology, size, and dissemination of oxygen species as the utmost appropriate surface defects^13^. This unique property of Ce^3+^ revealed high thermo-chemical robustness and large O_2_ species movement, and thus displays improved performance in catalytic oxidation of hydrocarbons and nitrogen oxides. So far, nonstoichiometric perovskite materials demonstrated some specific physical properties including evolution in surface defects, oxygen ion mobility, and redox property.

In this article, we proposed the synthesis of Ce^3+^ ion substituted LaMnO_3_ nanoparticles via sol-gel based co-precipitation process. We inspected the impact of Ce^3+^ ion doping in LaMnO_3_ nanoparticles on physiochemical properties and oxidation performance of C_6_H_5_CH_2_OH to C_6_H_5_CHO. For characterization various techniques were applied including X-ray diffraction pattern (XRD), transmission electron microscope (TEM), energy dispersive x-ray analysis (EDX), N_2_ adsorption, Fourier transform infrared (FTIR), optical absorption (UV-Vis), thermogravimetric analysis (TGA), temperature program reduction (TPR), temperature program oxidation (TPO) and X-ray photoelectron spectroscopy (XPS) techniques. These techniques revealed the role of Ce^3+^ ion substitution on the crystal structure, crystallinity, surface properties, thermal stability, optical, redox behavior, oxygen adsorption properties and catalytic activities of the as-prepared nonstoichiometric LaMnO_3_ materials.

## Experimental Section

### Synthesis of perovskites (La_1−x_Ce_x_MnO_3_)

Analytical grade chemicals were procured and used directly without any extra distillation. In a typical synthesis of LaMnO_3_ perovskite, 4.3 g La(NO_3_)_3_.6H_2_O (99.99%), and 2.4 g Mn(NO_3_)_3_.3H_2_O (99.99%, BDH Chemicals Ltd, UK), were dissolved in 50 ml H_2_O along with C_6_H_8_O_7_.H_2_O (E-Merck, Germany). Citric acid was used as a chelating agent for complexation with lanthanum and manganese nitrates. The resulting mixed aqueous solution was magnetically stirred on a hot plate at 100 °C until the transparent solution was achieved. Aqueous ammonia solution was quickly added to precipitation under constant mechanical stirring. The occurrence of the willing product was dried at 100 °C for overnight and further annealed at 700 °C in the air for 5 hrs. A similar procedure was repeated for synthesis of La_1−x_Ce_x_MnO_3_ oxides (x = 0.05, 0.07 and 0.10 mol %).

### Catalyst characterization

Powder X-ray diffraction measurement was performed on a PANalytical X’PERT (X-ray diffractometer) furnished with Ni filter and using Cu*Kα* (λ = 1.5406 Å). Morphology was obtained from Field emission Transmission Electron Microscope (FE-TEM, JEM-2100F JEOL, Japan) furnished with energy dispersive x-ray analysis (EDX) functioned at an accelerating voltage of 200 kV. Thermal analysis was measured on (TGA/DTA Mettler, Toledo, AG, Analytical CH-8603, Schwerzenbach, Switzerland). UV/Vis absorption spectra were measured by using Perkin-Elmer Lambda-40 Spectrophotometer. Fourier transforms Infrared (FT-IR) spectra were recorded on Perkin-Elmer 580B IR spectrometer. Temperature program reduction (TPR) and Temperature program oxidation (TPO) spectra were recorded on chemisorption Micromeritics AutoChem model 2910 analyzer furnished with a thermal conductivity indicator. Before the experiment, 100 mg material sample was treated with 10 vol % O_2_/He stream at 500 °C for 30 min to get complete oxidation. Then materials were cooled at room temperature and a mixture of 10 vol% H_2_/Ar gas with flow rate 20 mL/min was introduced and the reactor was heated from ambient temperature to 900 °C and maintained this temperature up to 20 min. For the O_2_- TPO experiments, helium(He, 30 mL/min) gas was applied for drying the perovskite samples at 150 °C and cooled down to room temperature, followed by an increase of temperature under O_2_/He (30 mL/min) flow with a temperature slope of 10 °C/min to 900 °C on the same instrument. The textural properties of the perovskites were recorded on a Micromeritics TriStar 3000 BET Analyzer, taking a value of 0.162 nm^2^ for the cross-sectional area of the N_2_ molecule adsorbed at 77 K. Powder samples were dried and degassed by heating gently to 90 °C for 1 h, then at 200 °C for 3 h under flowing N_2_ before measurement. The free space in each sample tube was determined with He, which was assumed not absorb.

### Catalytic studies

Liquid-phase oxidation of benzyl alcohol was carried out in a glass vessel equipped with a magnetic stirrer, reflux condenser, and thermometer. Briefly, a mixture containing benzyl alcohol (2 mmol), toluene (10 mL) and the perovskite (0.3 g) was vigorously stirred in a three-necked round-bottomed flask (100 mL) and then heated up to 120 °C. The O_2_-gas was introduce in the reaction mixture through bubbling to start the oxidation experiment with a 20 mL/min flow rate. After completion of reaction solid catalyst extracted from the solution by centrifugation and reaction mixture was analyzed by gas chromatography to examine the conversion of the alcohol and product selectivity by (GC, 7890 A) Agilent Technologies Inc, equipped with a flame ionization detector (FID) and a 19019S-001 HP-PONA column.

The specific activity of the catalyst was calculated using the equation

1 $$\begin{array}{rcl}{\rm{Specific}}\,{\rm{activity}} & = & {\rm{Moles}}\,{\rm{of}}\,{\rm{substrate}}\,({\rm{mmol}})\\  &  & \times {\rm{Product}}\,{\rm{formed}}/{\rm{Amount}}\,{\rm{of}}\,{\rm{catalyst}}({\rm{g}})\times {\rm{Reactiontime}}({\rm{h}})\end{array}$$

The turnover number and turnover frequency of the catalyst were calculated using

2 $${\rm{Turnover}}\,{\rm{numbers}}={\rm{Moles}}\,{\rm{of}}\,{\rm{desired}}\,{\rm{product}}\,{\rm{formed}}/{\rm{Number}}\,{\rm{of}}\,{\rm{active}}\,{\rm{centers}}$$

3 $${\rm{Turnover}}\,{\rm{frequency}}={\rm{turnover}}\,{\rm{number}}/{\rm{reaction}}\,{\rm{time}}$$

## Results and Discussion

### Crystallographic and morphological structure

**Figure 1:**
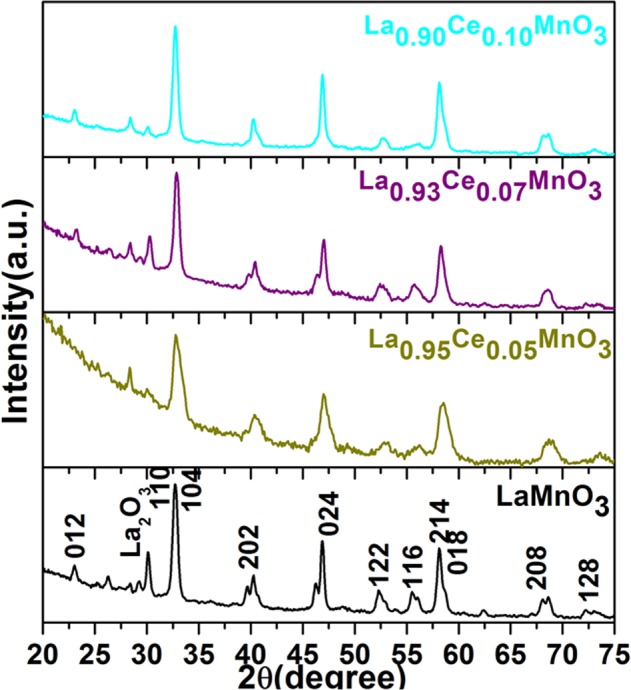
X-ray diffraction pattern of LaMnO_3_, La_0.95_Ce_0.05_MnO_3_, La_0.93_Ce_0.07_MnO_3_ and La_0.90_Ce_0.10_MnO_3_ nanoparticles.

Figure 1 demonstrates the XRD pattern to observe the chemical composition, crystallographic structure and grain size of the as-synthesized perovskite. As observed in Fig. 1. the distinct diffraction lines of perovskite in XRD pattern can be assigned to the (012), (110), (104), (202), (024), (122), (116), (214), (018), (208) and (128) lattice planes, which are attributed to the hexagonal structure of LaMnO_3_ nanoparticles(Fig. 1) (JCPDS card No. 032-0484)^6,19^. Any other diffraction line associated with MnO or CeO_2_ is not identified over the whole XRD range specifies the homogeneous dispersion into the crystal lattice and formation of perfect single phase LaMnO_3_ perovskite. An observed diffraction line at 30.27° corresponds to La_2_O_3_, which is weaker than the reflection lines of LaMnO_3_ perovskite. All diffractograms of the perovskite materials revealed the similar trigonal symmetry in the crystallographic space group with marginally dissimilar cell parameters. As shown in Fig. 1 diffraction lines in trivalent Ce^3+^ substituted perovskite are slightly shifted towards longer angle along with reduced intensity in respect to the un-substituted LaMnO_3_ perovskite, it could be due to the effect of Ce^3+^ ion doping into the crystal matrix. Owing to the small radius of Ce^3+^ ions, they are highly mobile and easily migrate from surface to crystal lattice within the crystal matrix of perovskite materials at environment conditions^13,14,20^. The broadening of reflection lines in perovskite materials suggested the nanocrystalline nature of the as-prepared nanomaterials. As shown in Fig. 1, on substituted of small radius Ce^3+^ (1.25 Å) in place of La(1.27 Å), the reflection lines slightly shifted to higher 2*θ*, signifying that the crystal arrangement becomes distorted^13,21^, resulting the transformation is occurring in the symmetry of crystallographic structure^7,10,22^. The experimentally calculated lattice parameters for LaMnO_3_, La_0.95_Ce_0.05_MnO_3_, La_0.93_Ce_0.07_MnO_3,_ and La_0.90_Ce_0.10_MnO_3_ are a = 5.527 Å, 5.463 Å, 5.449 Å and 5.436 Å, respectively, are decreased on increasing the substitution concentrations of the Ce^3+^ ion into the LaMnO_3_ crystal lattice in respect to un-substituted LaMnO_3_ perovskite. These variations in lattice parameters and shifts in peak positions endorse the substitution of modified ions into the crystal lattice structure.

**Figure 2:**
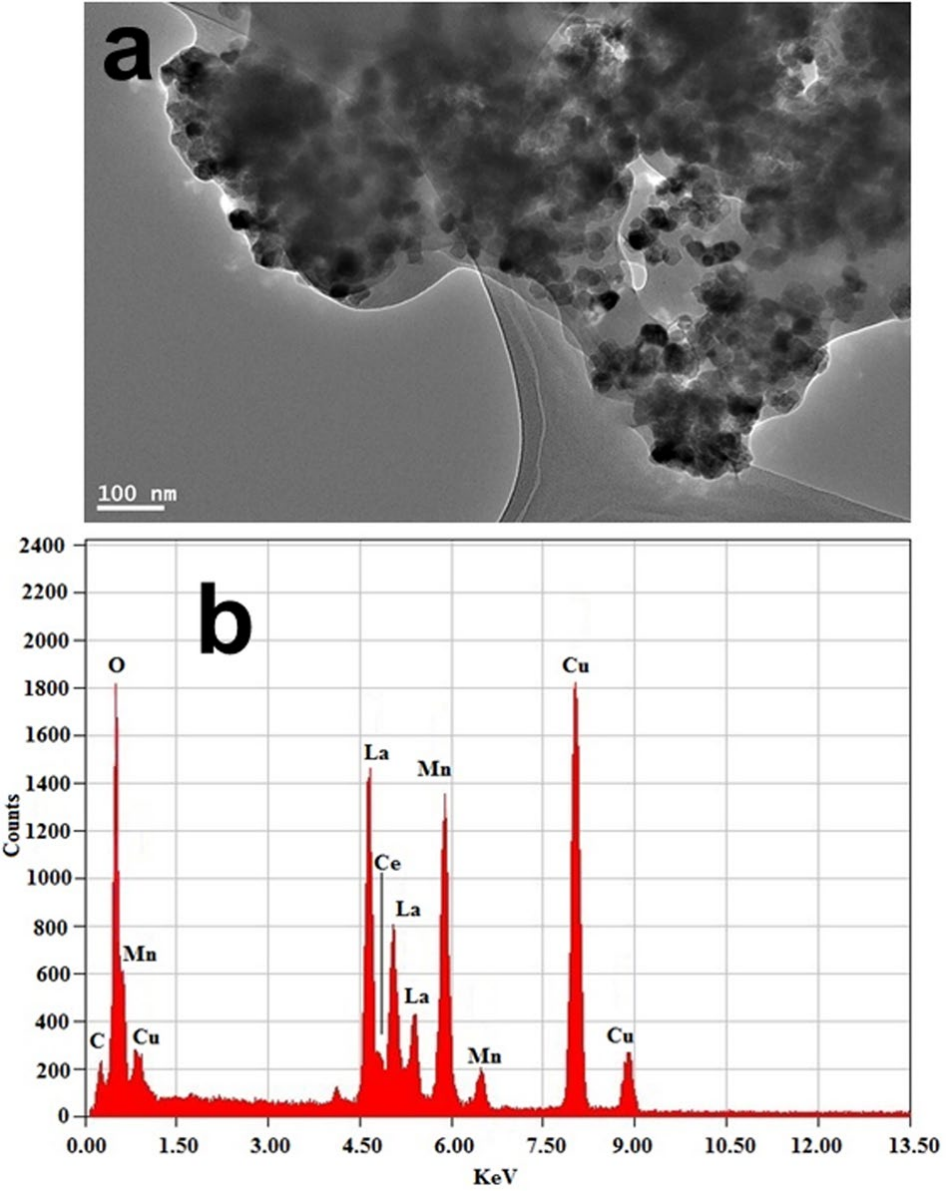
(**a**) TEM image and (**b**) EDX analysis of LaMnO_3_, nanoparticles.

TEM micrograph clearly shows the irregular hexagonal structure, smooth surface, uncontrolled size, highly aggregated, well-distributed nanoparticles. Figure 2a illustrates the typical image of Ce^3+^ ion substituted LaMnO_3_ perovskite nanoproduct with size ranging from 25–31 nm. Energy dispersive x-ray analysis in Fig. 2b revealed the existence of all substituted elements including La^3+^, Mn^3+^, Ce^3+^ and oxygen elements in the as-prepared LaMnO_3_ perovskite. The appearance of intense peaks of Cu^2+^ and C belong to the carbon coated copper grid. It confirmed the efficacious doping of Ce^3+^ into the crystal matrix.

### Textural properties and thermal stability

The structural parameters after calcination of Ce substituted LaMnO_3_ catalysts, Specific surface area (BET), pore volume (PV) and average pore size (PD) are summarized in Table 1. The PV and PD were obtained from the adsorption branch of the respective N_2_ isotherm by put on the BJH method. Surface area (Single point BET and Multipoint BET), PV and PD drop with increasing Ce ion concentrations from 5 to 10 mol% (Table 1).

**Table 1:** Textural properties of the Ce doped catalysts (La_1−x_Ce_x_MnO_3_).

Nominal Composition	Single point BET (m^2^/g)	Multi point BET (m^2^/g)	Pore volume (cm^3^/g)	Pore size (A)
LaMnO_3_	7.754	8.34	0.0013	18.63
La_0.95_Ce_0.05_MnO_3_	7.22	7.79	0.0011	18.61
La_0.93_Ce_0.07_MnO_3_	7.30	7.75	0.0012	18.60
La_0.90_Ce_0.10_MnO_3_	6.54	6.93	0.0011	18.59

**Figure 3:**
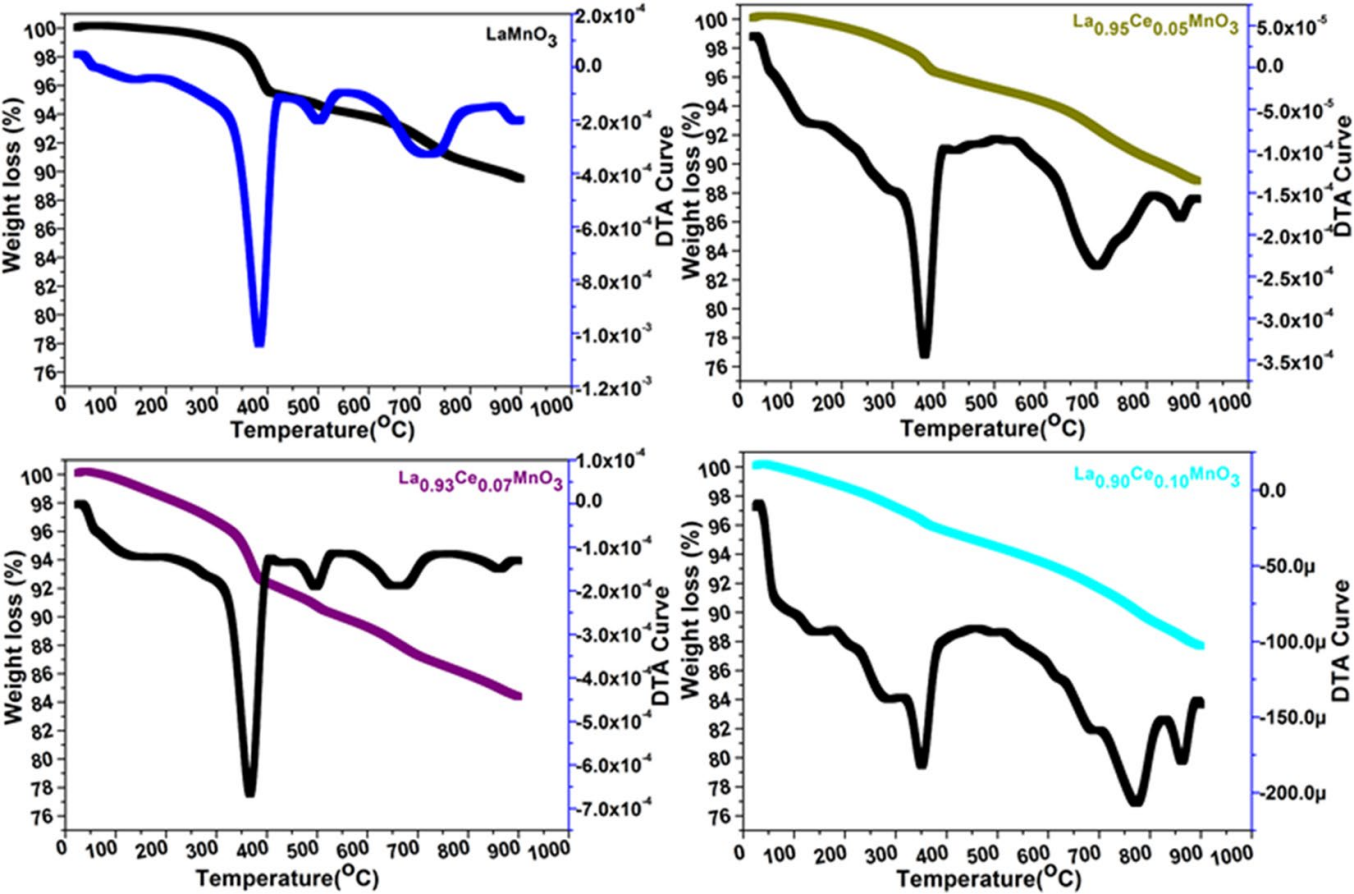
Thermogravimetric analysis of LaMnO_3_, La_0.95_Ce_0.05_MnO_3_, La_0.93_Ce_0.07_MnO_3_ and La_0.90_Ce_0.10_MnO_3_ nanoparticles.

Thermogravimetric (TGA) analysis of the as-prepared LaMnO_3_ perovskite and Ce-substituted materials exhibit a similar decomposition trend in all thermograms (Fig. 3). TGA spectra were recorded from 0–900 °C in N_2_-atmosphere with a heating rate of 10 °C/min (Fig. 3). First big exothermic peak (DTA) in all samples are observed at around 400 °C resemble the crystalline H_2_O molecules or complexation form surface attached organic impurities. The surface attached OH groups or organic moieties are coordinated to the central metal ion in different attachment form in the existing complex precursor system^23,24^. Generally, -OH groups attached on the surface of metal ions in two forms either terminal Ln-OH or in the bridge from Ln-(OH)-Mn^25^. In both cases, the dissociation of surface OH groups contrasts from each other depending on the surrounding chemical environment. So that, the reduction ii molar mass occurs in a rather varied range of temperature. No decomposition peaks signifying further crystallization are found in TGA, specifying that the perovskite materials are in crystalline form, as verified by XRD results. All four thermograms illustrate the sluggish weight loss (~6–8%) in between 400–900 °C, which is assigned to the removal or combustion of carbon dioxide at high temperature.

### Optical properties

**Figure 4:**
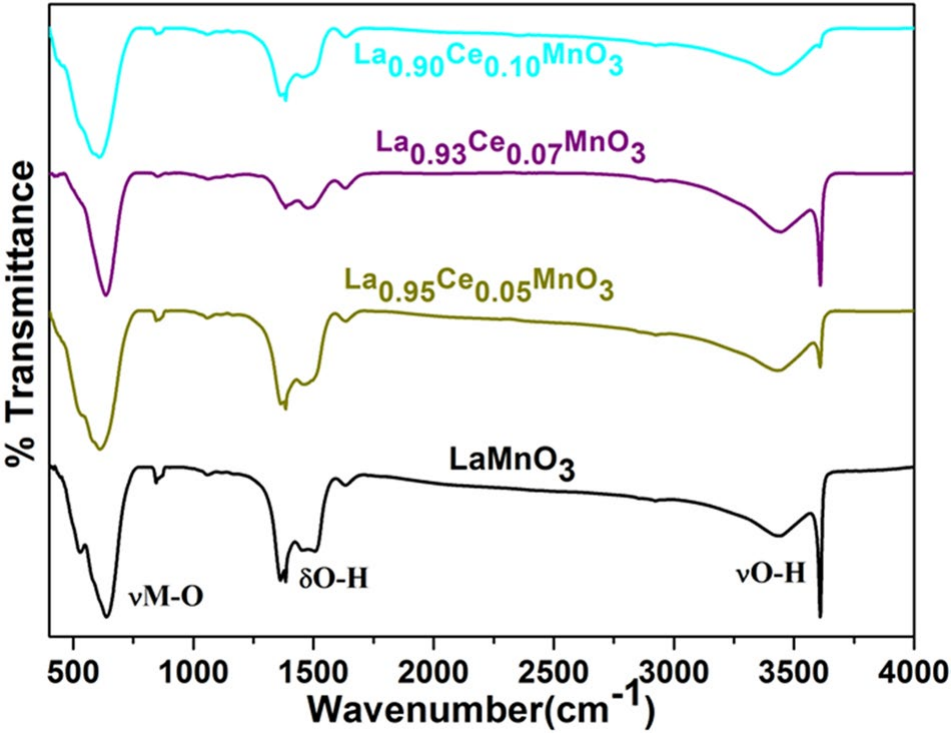
FTIR spectra of LaMnO_3_, La_0.95_Ce_0.05_MnO_3_, La_0.93_Ce_0.07_MnO_3_ and La_0.90_Ce_0.10_MnO_3_ nanoparticles.

Figure 4 displays the infrared spectra of the as-synthesized LaMnO_3_ and different Ce ion substituted LaMnO_3_ perovskite nanoparticles. All samples exhibited a diffused band in between 3160–3653 cm^−1^ assigned to the νO–H stretching vibration originating from surface adsorbed H_2_O molecules (Fig. 4)^25^. Two additional strong intensity infrared bands are observed positioned at 1486 and 1375 cm^−1^ attributed to the δOH and γOH vibrational modes of H_2_O molecules. These observed infrared spectral results are in accord with TGA observations. The observed infrared band at 644 cm^−1^ is allotted to the νM-O stretching vibrational mode which certified the formation of metal oxide framework^26,27^.

**Figure 5:**
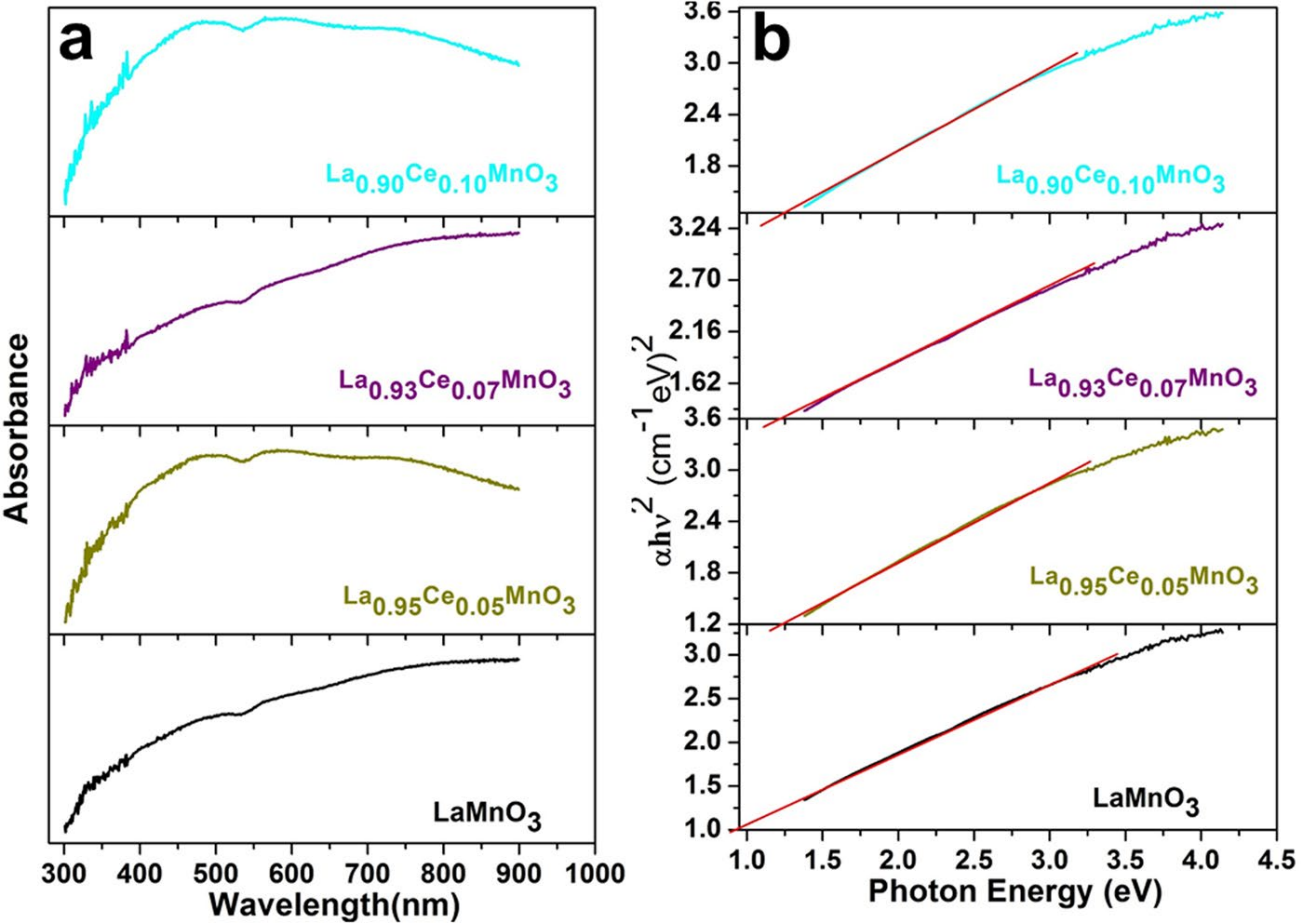
(**a)** UV/Vis absorption spectra and (**b**) The plot of (αh*ν*)^2^ vs. photon energy(h*ν*) LaMnO_3_, La_0.95_Ce_0.05_MnO_3_, La_0.93_Ce_0.07_MnO_3_ and La_0.90_Ce_0.10_MnO_3_ nanoparticles.

Optical absorption spectra were carried out to determine the optical characteristics of the as-synthesized perovskites (Fig. 5a,b). The direct energy band gap (*E*_*g*_) is estimated by fitting the absorption spectral data to the straight transition equation by extrapolating the linear portions of the curve into *αhν* = A(*hν* − *E*_*g*_)½, where *α* is optical absorption coefficient, *hν* is the photon energy, *E*_*g*_ is the direct bandgap and A is constant (Fig. 5b)^25,28,29^. The experimentally assessed direct energy band gaps of all perovskite nanomaterials are 1.15, 1.31, 1.34 and 1.32 eV for LaMnO_3_, La_0.95_Ce_0.05_MnO_3_, La_0.93_Ce_0.07_MnO_3_, and La_0.90_Ce_0.10_MnO_3_ perovskites, respectively. An observed increase band gap energy with increasing the Ce^3+^ ion substitution quantity into the LaMnO_3_ crystal lattice, which is attributable to the Burstein-Moss effect^28,30–32^.

### Redox properties (TPR/TPO)

**Figure 6:**
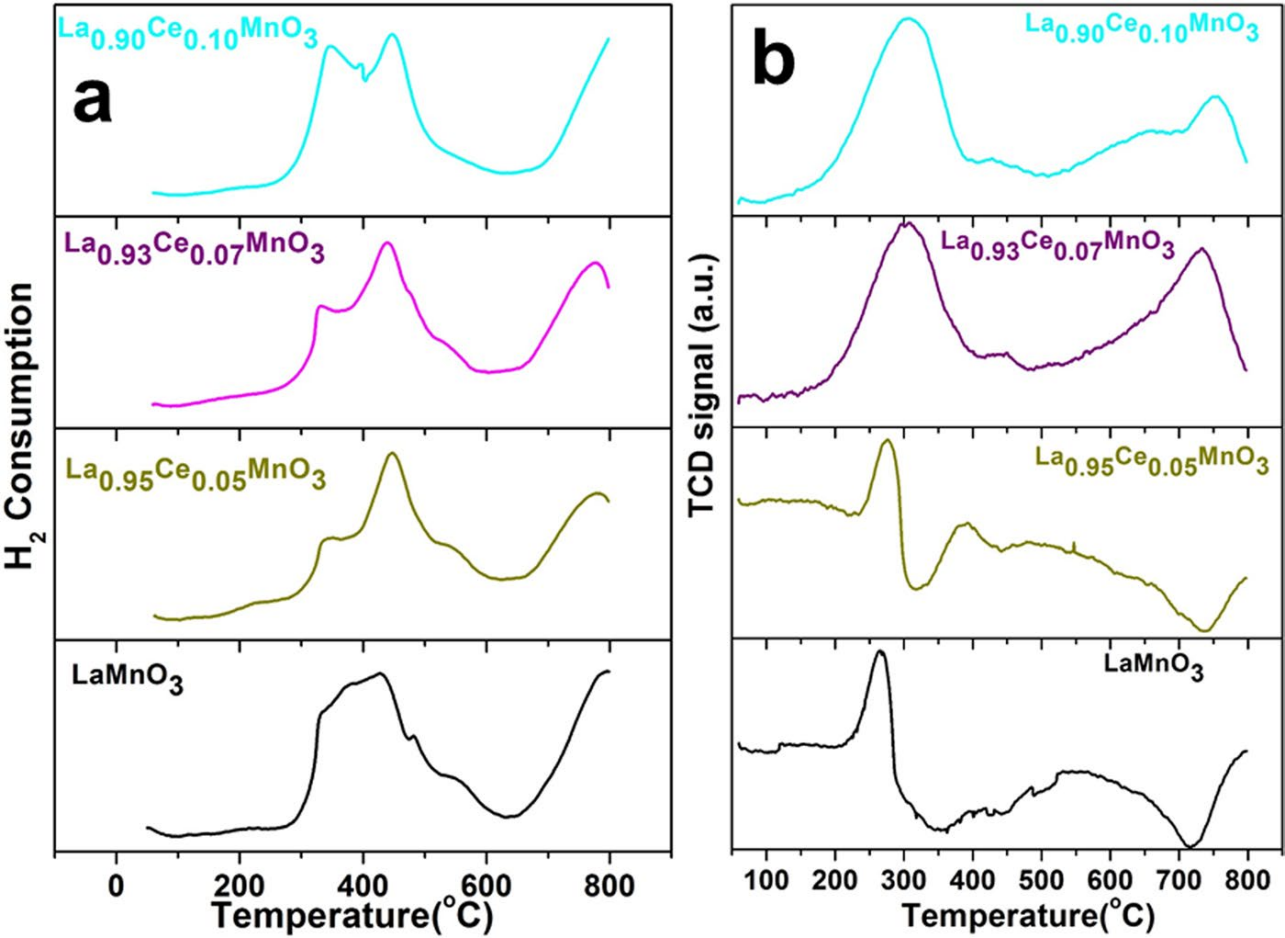
(**a)** Temperature program reduction and (**b**) Temperature program oxidation spectra of LaMnO_3_, La_0.95_Ce_0.05_MnO_3_, La_0.93_Ce_0.07_MnO_3_ and La_0.90_Ce_0.10_MnO_3_ nanoparticles.

Redox properties of the as-prepared LaMnO_3_ perovskite and their Ce^3+^ ion substituted LaMnO_3_ perovskites are determined by H_2_-TPR and the observed results are presented in Fig. 6a and tabulated in Table 2. TPR and TPO studies are performed to examine the role of Ce^3+^ ion-doping on redox behavior of LaMnO_3_ perovskite within the range from 50–800 °C. The TPR spectra were recorded within the temperature range from 50 to 800 °C temperature. TPR spectra exhibited two typical characteristic reduction peaks, first one in between 280–600 °C and second started from 645 °C^5^. The observed peak at low reduction temperature (280–600 °C) is correspond to the reduction of Mn^4+^ to Mn^3+^ and elimination of surface adsorbed oxygen vacancies, and the second reduction band is observed at a higher temperature (645 °C), which correspond to the reduction of Mn^3+^ to Mn^2+^ ^4,6,7,33,34^. The first broadband occurred at lower reduction temperature indicate the largest H_2_-consumption, it suggesting the better initiative catalytic activities of LaMnO_3_ perovskite at a lower temperature. The higher oxidation state of Mn^3+/4+^ ions is accountable for more oxygen species because of lacking ligand amounts of Mn^3+/4+^ ion. The occurrence of Mn^4+^ ion is associated with the fact that Mn^3+^ has a permitted electron, and have the ability to adsorb molecular O_2_ and convert it into an electrophilic form^6^. Reversed transformation of manganese ion oxidation states is observed by the TPO analysis (Fig. 6b), in which the oxidation peak at low temperature (205–310 °C) suggest the transition of Mn^2+^ to Mn^3+^ and the oxidation peak at 445–717 °C exhibit the oxidation from Mn^3+^ to Mn^4+^. These observations are in accord with published reports^4,5,34^.

**Table 2:** H_2_ consumption of La_1−x_Ce_x_MnO_3_ perovskite oxide.

Catalysts	Tmax1 (°C)	H_2_-uptake (cm³/g STP)	Tmax2 (°C)	H_2_-uptake (cm³/g STP)	Tmax3 (°C)	H2-uptake (cm³/g STP)	Total uptake (cm³/g STP)
LaMnO_3_	400	35.16	775.0	43.65			78.81
La_0.95_Ce_0.05_MnO_3_	343.3	11.54	447.0	72.13	775.6	2.24	85.91
La_0.93_Ce_0.07_MnO_3_	330.7	0.880	438.4	3.85	771.2	0.36	5.09
La_0.90_Ce_0.10_MnO_3_	345.6	98.51	450.2	17.40	895.8	84.31	200.22

Additionally, the H_2_-TPR profile shape of LaMnO_3_ is altered after doping of different Ce^3+^ ion concentrations into the LaMnO_3_ crystal lattice as seen in Fig. 6a. The incorporation of Ce^3+^ ion into the LaMnO_3_ matrix strongly modified the reduction behavior of LaMnO_3_ perovskite. As shown in Fig. 6a, the Ce^3+^ ions-substituted sample revealed three peaks at 330–345, 440–450 and ~800 °C, the first band looks very minute and the second band occurs very robustly^35^. The occurrence of two peaks in Ce^3+^ ion substituted LaMnO_3_ TPR profiles indicates the existence of at least two species in the LaMnO_3_ crystal lattice, which became stronger and shifted towards high temperature after increasing the doping concentrations of Ce^3+^. An observed band between 330–345 °C, ascribed to the replacement of Mn^2+^ by Ce^3+^ in LaMnO_3_ crystal matrix. Because of this charge disparity lattice alteration would arise that promote to the construction of La-O-Mn–O–Ce solid solution form, resulting the reactive O_2_ vacancies are produced that may be reduced simply at low temperature. Generally, the elimination of oxygen vacancies at low temperatures associated with higher oxygen mobility (oxygen reacts more easily) and oxygen reactivity^4,6^. An observed reduction band at 448 °C ascribed to the dissociation of powerfully interactive MnO_2_ type with Ce^3+^ supports, whereas weak intensity reduction band observed at ~800 °C consigned to the high-temperature dissociation band because of bulk MnO_2_ ^24^. Owing to the variation in balance of both metal (Mn^3+/4+^ and Ce^3+/4+^) cations from 4+ to 3+ or from 3+ to 2+, the up-down swings of O_2_ imperfections escorted with valence alteration is observed^6,35^. Therefore, the high O_2_ storage capacity of 10 mol% Ce substituted LaMnO_3_ perovskite because of the simultaneous occurrence of transportable O_2_ vacancies and analogous (Mn^2+/3+/4+^/Ce^3+/4+^) redox couples. Consequently, the La_0.90_Ce_0.10_MnO_3_ sample revealed an excellent catalytic activity at a lower temperature, so that, the highest redox properties, these results are in accord with previous literature reports^7,24,33^. Comparatively the intensity of the high-temperature components is remarkably varied on increasing the Ce ion concentrations, whereas peak positions (decomposition temperature) are almost similar. It suggested the similar type of species is reduced at the same temperature, which enhanced by Ce^3+^ ion substitution.

As shown in Fig. 6a, La_0.90_Ce_0.10_MnO_3_ sample revealed high reducibility at high temperature. So that, the replacement of La^3+^ by Ce^3+^ ion would effect in enhanced concentrations of Mn^3+^ ions and oxygen vacancies because of charge discrepancy accomplished by oxidation of Mn^2+^ to Mn^3+^ and by the construction of an oxygen-deficient perovskite La_0.90_Ce_0.10_MnO_3_, which would enhance the reducibility character of the perovskite. These observations are well consistent with XRD and XPS results, in which non-Ce ion substituted Mn^2+^ species are oxidized and transform into Mn^3+^ valence states. It inferred that the reducibility behavior of the perovskites in the following sequence LaMnO_3_ ≤ La_0.95_Ce_0.05_MnO_3_ ≤ La_0.90_Ce_0.10_MnO_3_ ≤ La_0.90_Ce_0.10_MnO_3_, according to the H_2_ consumption at 446 °C and 800 °C. Generally, oxygen species are attached with metal ion into two different bonding forms including non-crystalline and crystalline bonding forms. In the non-crystalline bonding form, the oxygen species are present in the outer coordination sphere and is referred to as surface adsorbed oxygen species. Whereas in case of crystalline bonding form, the oxygen species entered into the inner coordination sphere and compensate its valence state. These crystalline form oxygen species can be typically eliminated in metal oxide products at higher temperature^36,37^.

Temperature program oxidation or desorption was performed to evaluate the catalytic affinity towards oxygen. Figure 6b illustrates the TPO profile of the as-prepared LaMnO_3_ and different Ce^3+^ ion concentration substituted LaMnO_3_ perovskites. The TPO- profile of blank LaMnO_3_ perovskite in Fig. 6b, illustrate three oxygen desorption regions, at three different temperatures including 266, 533 and ~799 °C, respectively. An observed first band at 266 °C is attributed to the weakest oxygen vacancies (superficial O_2_ species), which are physiochemically adsorbed/chemisorbed O_2_ species and are eliminated at low-temperature. The appearance of broadband between 350–725 °C assigned to the non-stoichiometric oxygen (interfacial oxygen) vacancies and reduction of Mn^4+^ to Mn^3+^, which are desorbed at high temperature. Whereas the oxygen vacancies desorbed at a higher temperature (≥725 °C) can be attributed to the relocation of lattice O_2_ in the bulk perovskite phase and reduction of Mn^3+^ to Mn^2+^ ^7,10,33,35^. Generally, surface adsorbed O_2_ vacancies desorbed at low temperatures and interfacial oxygen in non-stoichiometric form desorbing at high temperature^23–25,33,35,36,38^.

As seen in Fig. 6b, when the Ce^3+^ ion is replaced in the La^3+^ site of LaMnO_3_ perovskite a charge balance is desired to attain the neutrality of the perovskite. It can either achieved by O_2_ defects or the swing of the Mn ion towards higher valance states (Mn^3+^ to Mn^4+^). As illustrated in Fig. 6b, on the substitution of 5 mol% Ce^3+^ ion doping the strong low-temperature peak is shifted towards slightly higher temperature, which corresponds to surface desorbed oxygen species. While high-temperature peak assigned to interfacial oxygen species is split into two peaks observed at 390 and 490 °C. However, on increasing the substitution concentration of Ce^3+^ ion in LaMnO_3_ crystal lattice, the low temperature desorption peaks are moved towards higher temperature with significant enhanced integral area, indicating the homogeneous substitution of Ce^3+^ ion into crystal lattice which increase the oxygen ion mobility of both surface (superficial) oxygen species and non-stoichiometric (interfacial) lattice oxygen species, it could be due to the effect of small ionic size Ce^3+^ ion substitution^13,24,25^. As observed previously, the Ce^3+/4+^ ions have high oxygen species motilities because of their multiple oxidation states. The high-temperature O_2_ desorption of LaMnO_3_ is typically denoted to as the removal of non-stoichiometric surplus oxygen. It could be due to the creation of Mn^3+^ in LaMnO_3_ to reduce the Jahn–Teller distortion, although the charge stability advocates that Mn should be in 3+ oxidation state. In La_0.90_Ce_0.10_MnO_3_ the Mn^3+^ state is highly stable because of the existence of Ce^3+^ ions in the crystal lattice (charge compensation)^33^.

### XPS studies

**Figure 7:**
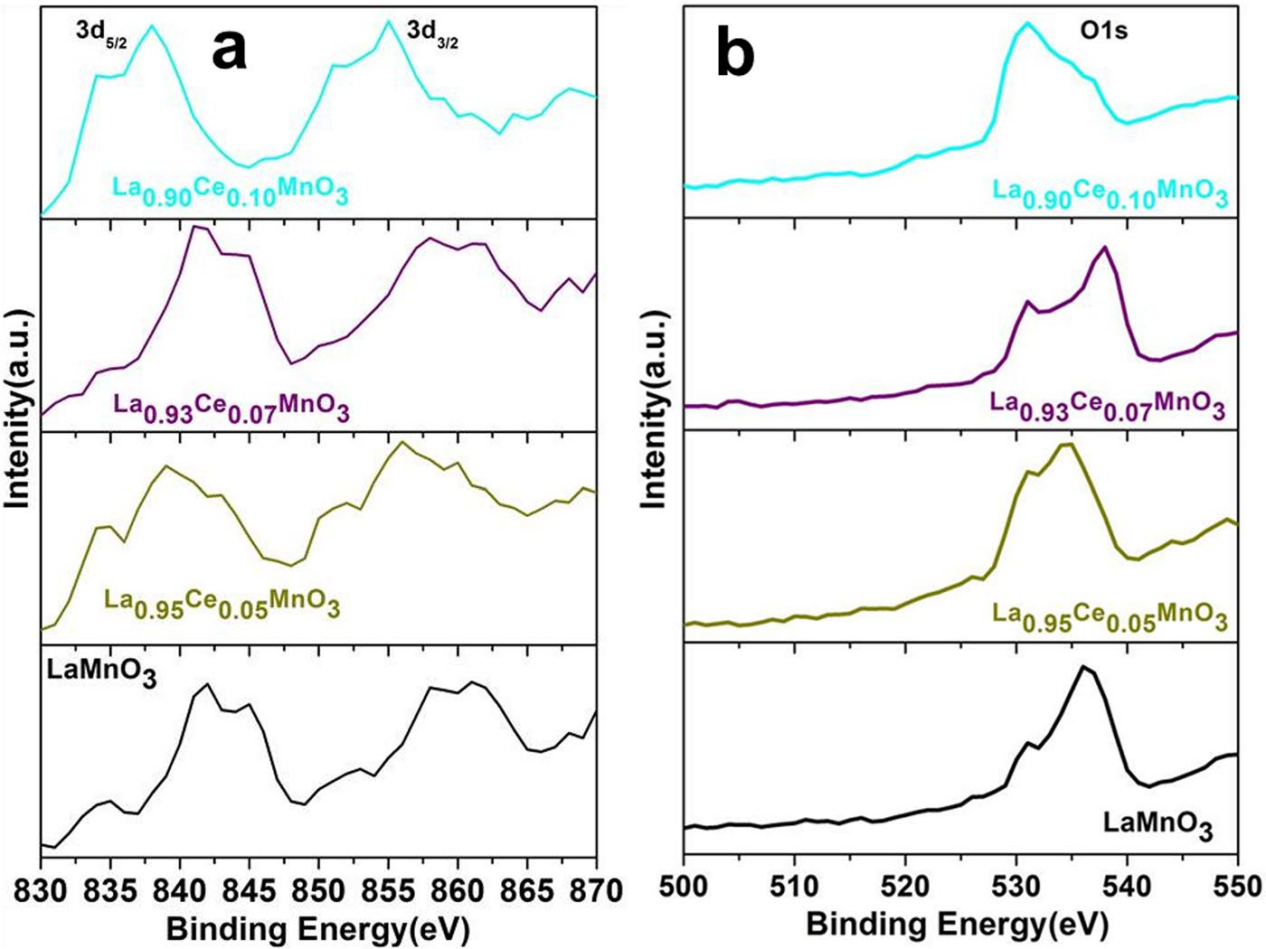
(**a**) XPS analysis of the La 3d_3/2&5/2_ and (**b**) O1s spectra recorded for the LaMnO_3_, La_0.95_Ce_0.05_MnO_3_, La_0.93_Ce_0.07_MnO_3_ and La_0.90_Ce_0.10_MnO_3_ nanoparticles.

**Figure 8:**
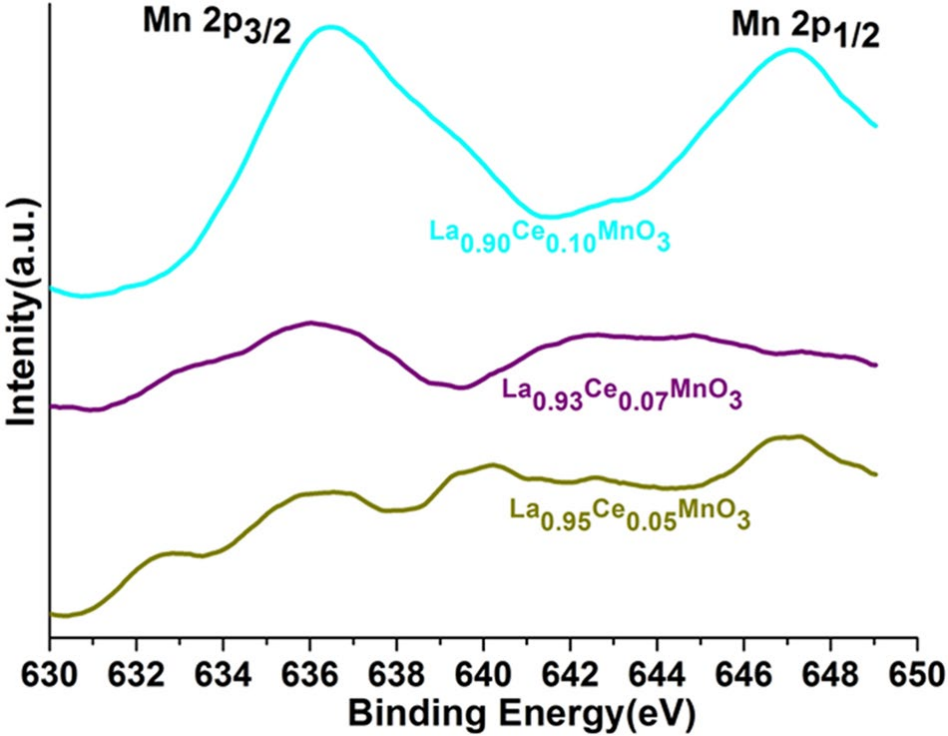
XPS analysis of the Mn 2p_1/2&3/2_ spectra recorded for the La_0.95_Ce_0.05_MnO_3_, La_0.93_Ce_0.07_MnO_3_ and La_0.90_Ce_0.10_MnO_3_ nanoparticles.

The surface chemical components, phase purity, and their oxidation states are inspected by XPS analysis. Figures 7 and 8 demonstrated the XPS spectra of La(3d & 4d), Mn(2p) and O(1 s) for the different Ce ion concentration substituted perovskites. XPS spectra of the La 3d in the LaMnO_3_ and La_x_Ce_1−x_MnO_3_ displayed two binding energies (BE) bands located at 844 and 860 eV which correspond to the La 3d_5/2_ and La 3d_3/2_, respectively. The existence of these valence band indicates that lanthanum in La^3+^ ion form(Fig. 7a)^1^. Additionally, each band has additional satellite band along with core band, owing to the relocation of electrons from O2p to the vacant orbital of La 5 f orbital. These observations are similar to the previous values observed for La_2_O_3_ ^1,39^, it suggested the trivalent state of La^3+^ ions in the perovskite materials. The increased La 4d binding energy is interpreted as due to the displacement of the electron density toward nearest neighbors. The oxygen (O1s) signal in XPS spectra shows two peaks, the first one is centered at 531 eV and second at around 436 eV in La_0.95_Ce_0.05_MnO_3_ sample (Fig. 7b). As shown in Fig. 7b, the low BE band is due to the lattice oxygen, whereas broader band with high BE band is associated with the surface adsorbed oxygen or surface hydroxyl groups. Peng *et al*. observed that the surface adsorbed O_2_ is the most active oxygen because of higher mobility in respect of lattice oxygen, which plays a crucial role in conversion process through migration from the surface to lattice sites^1,3,13^.

As seen in Fig. 7b, on increasing the dopant concentration (Ce^3+^ ions) the peaks are varied along with broadening, it indicates the existence of several types of oxygen vacancies such as oxygen of hydroxyl (–OH^−^)/carbonate(–CO_3_^2−^) groups on the surface of matrices^2,7,8,10^ and it is in accord with the TPO results. According to the TPO results the observed low-temperature desorption band(surface O_2_ species) is directly related to the quantity of O_2_ species are in very small, while the high quantity of O_2_ species evolved at a higher temperature(chemisorbed O_2_ species). An observed an increase in core-level binding energy indicates that all of the cations in the samples (La, Ce, and Mn) are bonded to the oxygen. Most importantly, we are unable to observe the Ce ion peak in the current perovskites matrixes due to the Ce ion in LaCeMnO_3_ perovskites are mostly in the tetravalent state^40^.

An observed XPS peak located at around 655 eV is assigned to 2p_1/2_ of Mn ions, although the band of Mn 2p_3/2_ is composed of multiple bands it implies the presence of multivalence states such as Mn^2+^ (641), Mn^3+^(644) and Mn^4+^(648) (Fig. 8)^41–46^. Qureshi *et al*. observed that the splitting in Mn 2p peak is due to the asymmetric nature of the metal, which suggests Mn exists in the mixed valence state^46,47^. However, satellite structure at higher BE divided by ~4 eV, it could be due to the strong columbic interaction in between hybridization of Mn 3d electrons and other valence sub-shells^42,44,47^. No Mn 2p_3/2_ band for Mn (~639 eV) is detected in the spectrum, it implies that no metallic form of Mn is presented in the as-prepared perovskites (Fig. 8). The impact of the catalytic activity on MnOx is related to its oxidation states which are MnO_2_ > Mn_2_O_3_ > MnO as reported by Thirupathi & Smirniotis^4,10,48,49^. According to them, MnO_2_ is a highly reactive compound in all Mn-based compounds including MnO_2_, Mn_5_O_8_, Mn_2_O_3_, and Mn_3_O_4_. Therefore, Mn^4+^ has higher catalytic performance, and this resembled the finest catalytic denitration activity of La_90_Ce_10_MnO_3_. The peaks of the Mn 2p_1*/*2_ and Mn 2p_3*/*2_ of the applied materials are moved towards longer BE, observed at ~2 eV and 3 eV, respectively. As shown in Fig. 8, the binding energies are significantly varied upon increasing the Ce ion concentration into the perovskite matrix, it indicates the variation in valence states of Mn ions.

### Catalytic reaction

**Figure 9:**
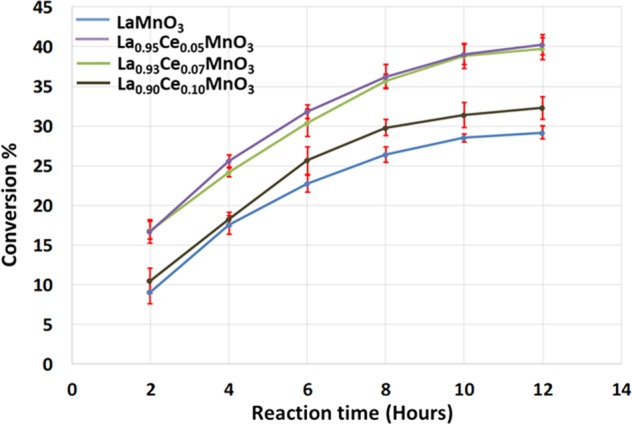
Graphical illustration of the kinetic of the reaction carried out using La_1−x_Ce_x_MnO_3_ catalysts. Conditions: catalyst = 0.3 g, T = 393 K, benzyl alcohol = 2 mmol, toluene = 10 mL, O_2_ flow rate = 10 cm^3^min^−1^, reaction time = 12 h.

**Figure 10:**
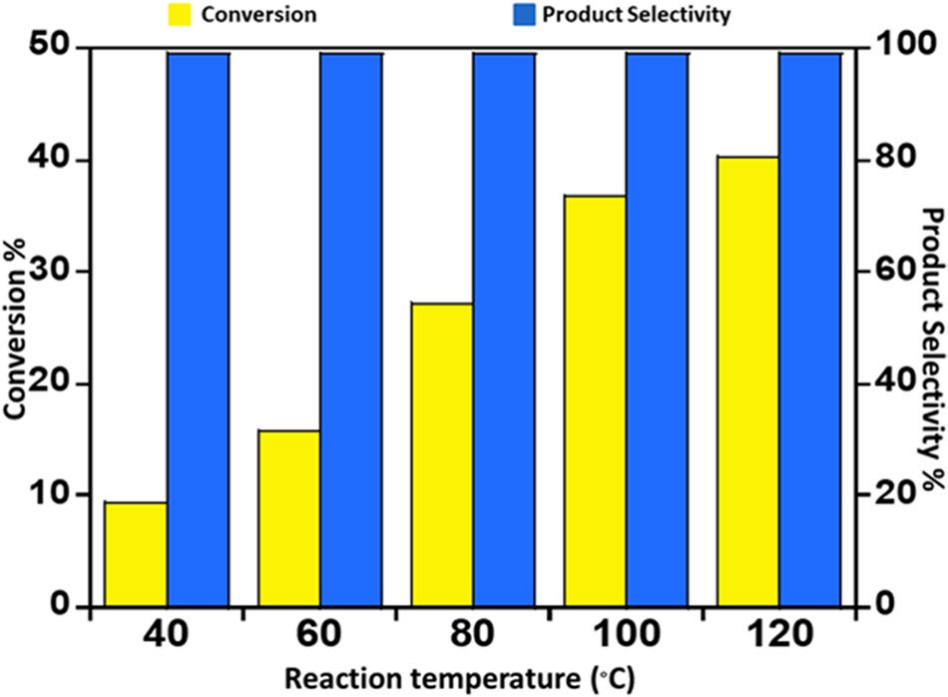
Graphical illustration of catalytic activity of La_1−x_Ce_x_MnO_3_ as a function of reaction temperature.

The prepared materials were exposed to catalytic assessment and the conversion of benzyl alcohol into benzaldehyde is taken up as a typical reaction. It was observed that the prepared catalysts are active against the substrate benzyl alcohol. Adding Ce in the LaMnO_3_ catalyst is found to impact on catalytic aerobic oxidation of benzyl alcohol due to the synergetic effect between Ce^3+/4+^ and Mn^3+/4+^ ions. The C_6_H_5_CHO is the core constituent, with an insignificant quantity of C_6_H_5_COOH as a byproduct. The perovskite LaMnO_3_ is found to yield a 29% benzaldehyde within 12 hours, while conversion yield is improved on increasing the Ce ion substitution concentration in the perovskite, as shown in Table 3 (Fig. 9). As demonstrated in Fig. 9, on the substitution of 0.05% Ce in the La_0.95_Ce_0.05_MnO_3_ catalyst yielded 10% more benzaldehyde i.e. 40% which is better than their parent or blank perovskite. Further modification of the catalyst with further increase in the percentage content of Ce in the catalytic system, yielded La_0.93_Ce_0.07_MnO_3_ and La_0.9_Ce_0.1_MnO_3_ respectively, it indicates that the catalytic activity decreases as the % of Ce^3+^ ion concentration increase in the catalyst composition. The catalyst La_0.93_Ce_0.07_MnO_3_ and La_0.9_Ce_0.1_MnO_3_ yielded 37% and 32% oxidation product, i.e. benzaldehyde, respectively. Furthermore, the selectivity towards benzaldehyde was found to be >99% in all the cases. The graphical representation of the results obtained for all the catalysts tested is given in Fig. 9. When the catalytic activity is compared to the external area of the as-synthesized perovskite, it was observed that the catalyst La_0.95_Ce_0.05_MnO_3_ which displayed the best catalytic performance has a surface area of 7.7922 m^2^/g, and it found to be lower than the surface area of the perovskite LaMnO_3_ i.e. 8.3410 m^2^/g, which yielded a 29% benzaldehyde within 12 hours lower than the catalyst La_0.95_Ce_0.05_MnO_3_ which yielded a 40% benzaldehyde. However, as the % of Ce in the catalyst composition is increased in the perovskites i.e. La_0.93_Ce_0.07_MnO_3_ and La_0.9_Ce_0.1_MnO_3_ the surface area further decreases to 7.7554 and 6.9371 respectively and the catalytic performance also depreciates. This indicates that the catalytic activity is not only dependent on the specific surface area it also depends on the doping concentration of the Ce^3+^ ion in the materials. An un-doped perovskite possesses Mn in +3 state, while upon the inclusion of the Ce^3+^ ions and the Mn oxidation state +4 (excess) and +2 is obtained as indicated by the XPS. Noticeably, Ce^3+^ ion concentration plays a crucial part in the enhancement of the catalytic performance as it induces a high surface oxygen mobility than their un-doped perovskite, and the Mn oxidation state +4 (excess) and +2 is obtained, which enhances the surface redox properties of the perovskites as confirmed by the XPS. However, further increase of the Ce^3+^ ions in the perovskite was found to result in the diminution in the catalytic performance, it specifies may be the depreciation in Mn^4+^ and Mn^2+^ sites and increase in the Mn^3+^ ion. Apart from the oxidation states of Mn, the decrease in the La^3+^ which results due to the increase of Ce^3+^ in the catalytic system may also be accountable for the depreciation in the catalytic activity. The specific catalytic activity of the as-designed materials is calculated based on the turnover number and turnover frequency as presented in Table 3. From the values obtained, it is found that the catalyst La_0.95_Ce_0.05_MnO_3_ has the highest TON and TOF among all the catalysts prepared. Further studies are determined in order to optimize the reaction temperature for the best catalytic performance, the catalyst La_0.95_Ce_0.05_MnO_3_, is utilized for the oxidation of C_6_H_5_CH_2_OH at various temperatures ranging from 40 °C to reflux temperature, and it was found that the catalyst performance is best at the reflux temperature, while at other temperatures, a slight decrease in catalytic performance was observed, observed results are illustrated in Fig. 10.

**Table 3:** Aerobic oxidation of benzyl alcohol employing La_1−x_Ce_x_MnO_3_ catalysts.

Entry	Catalyst	Conv. (%)	Sel. (%)	TON	TOF (h^−1^)
1	LaMnO_3_	29.18	>99	/	/
2	La_0.95_Ce_0.05_MnO_3_	40.25	<99	1127.92	93.99
3	La_0.93_Ce_0.07_MnO_3_	36.71	<99	734.79	61.23
4	La_0.9_Ce_0.1_MnO_3_	32.27	<99	450.15	37.51

## Conclusions

We successfully synthesized and characterized the Ce^3+^ ion substituted lanthanum magnetite perovskites materials by co-precipitation method and applied for conversion of benzyl alcohol into benzaldehyde. Chemical composition and phase purity of the as-synthesized materials were validated from XRD, EDX, TGA and FTIR analysis. The values of optical energy band gaps were varied because of discrepancy in the grain size of the perovskite materials. The increase in doping quantity of Ce^3+^ ions altered the redox (TPR and TPO) behavior of the perovskite oxides. The insertion of co-dopant Ce^3+^ ion in perovskite lattice enhanced the quantity of Mn^4+^ and chemisorbed oxygen positions on the surface of perovskite lattice to increase the catalytic performance. The XPS spectra of La 3d, Mn 2p, and O 1 s clearly revealed the influence of Ce ion substitution, which confirms the transformation of the Mn oxidation state from 3+ to 4+ due to the substitution of trivalent Ce^3+^ ions at the La^3+^ site in LaMnO_3_ perovskite. The surface Ce^3+^ ion in the perovskite matrix simplifies in oxidation and reduction of oxygen species which stimulates the oxy-dehydrogenation of benzyl alcohol to benzaldehyde. The Mn 2p_3/2_ core level XPS analysis suggests that due to oxygen vacancies, Mn^2+^ ions were generated from the Mn^3+^ transformation in perovskites. It is observed that La_0.95_Ce_0.05_MnO_3_ catalyst shows the highest TON and TOF among all prepared perovskites. According to our observed results the Ce^3+^ ion -doped LaMnO_3_ materials could serve as potential heterogeneous catalysts for hydrocarbon conversion. Besides that, trivalent cerium ion doping stimulate the synergistic effect within the crystal lattice along with different transition metal ions as co-catalysts to enhance the performance of the heterogeneous Fenton/perovskite process, an interesting point that merits further investigation.
